# Serum triglyceride and cholesterol concentrations and lipoprotein profiles in dogs with naturally occurring pancreatitis and healthy control dogs

**DOI:** 10.1111/jvim.15715

**Published:** 2020-02-03

**Authors:** Panagiotis G. Xenoulis, Paul J. Cammarata, Rosemary L. Walzem, Jan S. Suchodolski, Jörg M. Steiner

**Affiliations:** ^1^ Gastrointestinal Laboratory, Department of Small Animal Clinical Sciences College of Veterinary Medicine and Biomedical Sciences, Texas A&M University College Station Texas; ^2^ Clinic of Medicine, Faculty of Veterinary Medicine University of Thessaly Karditsa Greece; ^3^ Laboratory for Cardiovascular Chemistry, Department of Chemistry Texas A&M University College Station Texas; ^4^ Department of Poultry Science and Graduate Faculty of Nutrition Texas A&M University College Station Texas

**Keywords:** canine, inflammation, lipids, pancreas, ultracentrifugation

## Abstract

**Background:**

Previous studies have reported an association between hyperlipidemia and pancreatitis in dogs, but details of this association remain poorly defined.

**Hypothesis/Objectives:**

To compare serum triglyceride and cholesterol concentrations and lipoprotein profiles between dogs with naturally occurring pancreatitis and healthy dogs.

**Animals:**

Seventeen dogs with a clinical diagnosis of pancreatitis (Group 1) and 53 healthy control dogs (Group 2).

**Methods:**

Prospective case‐control study.

**Results:**

In Group 1, 3/17 dogs (18%) had hypertriglyceridemia whereas in Group 2, 4/53 dogs (7.5%) had hypertriglyceridemia (odds ratio [OR], 2.63; 95% confidence interval [CI], 0.52‐13.14; *P* = .35). A significant difference was found in serum triglyceride concentrations between Group 1 (median, 67 mg/dL) and Group 2 (median, 54 mg/dL; *P* = .002). In Group 1, 4/17 dogs (24%) had hypercholesterolemia, whereas 1/53 (1.9%) dogs in Group 2 had hypercholesterolemia (OR, 16; 95% CI, 1.64‐155.5; *P* = .01). No significant difference was found in serum cholesterol concentrations between Group 1 (median, 209 mg/dL) and Group 2 (median, 227 mg/dL; *P* = .56). Lipoprotein profiles were significantly different between Group 1 and Group 2 dogs (Eigenvalues, 0.6719; *R* ^2^ = 1.0; *P* = .001).

**Conclusions and Clinical Importance:**

Most dogs with pancreatitis (>70%) had serum triglyceride and cholesterol concentrations within reference intervals. In the small percentage of dogs that had hypertriglyceridemia, hypercholesterolemia, or both, increases were mild. Important differences were identified in lipoprotein profiles between dogs with pancreatitis and healthy control dogs. Dogs with pancreatitis had higher low‐density lipoprotein fractions and lower triglyceride‐rich lipoprotein and high‐density lipoprotein fractions than healthy dogs.

**Abbreviations**
BCS: body condition score
cTSH: canine thyroid‐stimulating hormone
HDL: high‐density lipoproteins
LDL: low‐density lipoproteins
NaBiEDTA: bismuth sodium ethylenediaminetetraacetic acid
Spec cPL: specific canine pancreatic lipase
T4: thyroxin
TRL: triglyceride‐rich lipoproteins
VLDL: very low‐density lipoproteins

## 1 INTRODUCTION

The nature of the association between hyperlipidemia and pancreatitis remains unclear in dogs, but it has been speculated to be bidirectional.^1^, ^2^ Hypertriglyceridemia, a form of hyperlipidemia, has been considered to be the cause of pancreatitis in some cases. This hypothesis is supported by the results of 2 clinical studies in dogs,^3^, ^4^ and is further supported by ex vivo studies^5^ in dogs and clinical studies in humans.^6^, ^7^, ^8^ On the other hand, hyperlipidemia also has been hypothesized to be the result of pancreatitis.^1^, ^2^ Although this association is widely believed to be true, scientific evidence supporting this hypothesis has not been documented in dogs with naturally occurring pancreatitis. Hypertriglyceridemia has been reported in dogs commonly with pancreatitis, but it remains unclear in these studies whether hypertriglyceridemia was the result of pancreatitis, a coexisting disease, a postprandial state, or a combination of these factors.^9^, ^10^, ^11^, ^12^, ^13^ Furthermore, with the exception of the results of 1 study,^14^ hypertriglyceridemia has not been reported to be a consequence of experimentally induced pancreatitis in dogs.^10^, ^15^, ^16^ However, experimental models of pancreatitis do not always replicate the pathophysiologic mechanisms of spontaneous disease.

Studies specifically investigating serum lipid concentrations and lipoprotein profiles in dogs with spontaneously occurring pancreatitis have not been reported. In addition, as stated above, reported studies in dogs with naturally occurring pancreatitis had certain limitations that do not allow for appropriate conclusions regarding this association. Characterization of the lipid concentrations and lipoprotein profiles of dogs with pancreatitis might clarify the association between hyperlipidemia and pancreatitis. Our aims were (1) to measure serum triglyceride and cholesterol concentrations in dogs with naturally occurring pancreatitis and compare them with results in healthy control dogs, and (2) to evaluate the lipoprotein profiles of dogs with naturally occurring pancreatitis and compare them with those of healthy control dogs.

## 2 MATERIALS AND METHODS

### 2.1 Dogs

#### 2.1.1 Group 1 (dogs with pancreatitis)

Requirements for inclusion of dogs in Group 1 were: (1) a clinical diagnosis of pancreatitis; (2) absence of diseases that can cause secondary hyperlipidemia (eg, hypothyroidism, hyperadrenocorticism)^1^, ^2^; (3) not receiving or having received any medications known to affect lipid metabolism (eg, glucocorticosteroids) for at least 3 months before inclusion in the study^1^, ^2^; (4) being fed diets not labeled as “low‐fat”; (5) withholding of food for at least 12 hours before blood collection; and (6) having enough serum for additional testing.

The database of the Gastrointestinal Laboratory at the College of Veterinary Medicine and Biomedical Sciences at Texas A&M University was searched for dogs with a serum Spec cPL concentration ≥400 μg/L (the currently recommended cut‐off value for pancreatitis).^17^, ^18^ Serum samples from dogs that were considered for inclusion into the study had been submitted to the Gastrointestinal Laboratory at Texas A&M University by veterinarians located throughout the United States as part of their diagnostic evaluation, and were stored at −80°C until further analysis. Dogs were recruited over a 6‐month period, but serum lipid concentrations and lipoprotein profiles were determined within 2 weeks after sample collection. Submitting veterinarians were contacted by phone and asked to complete a standardized questionnaire for each dog. Questions covered date of birth, sex and neuter status, body weight and body condition score (BCS), current diet, current medications, clinical presentation, and current and past health history of the dogs. Questionnaires from all dogs were reviewed to determine whether dogs fit the inclusion criteria for the study. Dogs were enrolled on a sequential basis based on whether the submitting veterinarian had agreed to fill out the questionnaire and whether the dogs fit the inclusion criteria. No preference was given to lipemic samples.

The diagnosis of pancreatitis was based on: (1) presence of historical findings of at least 2 of the following clinical signs: vomiting, anorexia, lethargy, abdominal pain, or diarrhea; (2) exclusion of other likely causes for these clinical signs based on additional testing (see below and Table 1) and follow‐up (acceptable follow‐up was considered discharge from the hospital [if hospitalized], substantial improvement with treatment for pancreatitis alone, or both); and (3) serum Spec cPL concentration ≥400 μg/L (the currently recommended cut‐off value for pancreatitis).^17^, ^18^ Routine diagnostic screening, including CBC, serum biochemistry profile with electrolytes, and thyroid profile (ie, total thyroxine [T4] and canine thyroid‐stimulating hormone [cTSH] concentrations and free T4 concentration by equilibrium dialysis) was performed to evaluate dogs for the possibility that hyperlipidemia was secondary to other conditions (eg, hypothyroidism, diabetes mellitus, hyperadrenocorticism). Based on the historical information for each dog and results of the tests performed, secondary causes of hyperlipidemia were excluded with reasonable certainty. Although dogs with concurrent diseases or those receiving medications that might affect lipid metabolism initially were included in this group to determine the overall prevalence of hyperlipidemia, they were later excluded from the analysis and ultimately only dogs that had pancreatitis without any other recognized risk factors for hyperlipidemia were included in this group.

**Table 1:** Characteristics of dogs with pancreatitis and healthy control dogs included into the study

Group characteristics	Pancreatitis	Healthy	*P* value
Total number, n	17	53	‐
**Patient characteristics**			
Age in years, mean (±SD)	7.4 (4.3)	5.4 (2.6)	.07
Sex, male/female	8/9	27/26	1
Neutered	16/17	46/53	.67
BCS, median (range)	5 (4‐6)	5 (4‐6)	.7
BCS category, n (%)			.67
≤5	16 (94%)	46 (87%)	
>5	1 (6%)	7 (13%)	
**Selected clinicopathologic variables (median [range])**			
Serum triglyceride (mg/dL)	67 (48‐324)	54 (26‐257)	**.002**
Serum cholesterol (mg/dL)	209 (142‐849)	227 (97‐338)	.57
Serum glucose (mg/dL)	108 (87‐138)	99 (85‐123)	.51
Serum total T4 (μg/d)	2.5 (1.3‐4.9)	2.1 (1.2‐5.2)	.43
Serum cTSH (ng/mL)	0.28 (0.09‐1.21)	0.22 (0.029‐0.96)	.74
Serum free T4 (ng/dL)	1.28 (0.6‐3.8)	1.4 (0.4‐2.3)	.62
Serum Spec cPL (μg/L)	642 (456‐1001)	29 (29‐186)	**<.0001**

*Note*: Bold face values indicate statistical significance at *P* < .05.

Abbreviation: BCS, body condition score (range of possible scores: 1‐9).

#### 2.1.2 Group 2 (healthy control dogs)

Inclusion criteria for the control group were: (1) absence of any clinical signs at the time of blood collection and the preceding 2 months, (2) no major abnormalities on the serum biochemistry profile, (3) a serum Spec cPL concentration within the reference interval, (4) absence of diseases that may cause secondary hyperlipidemia (eg, diabetes mellitus, hypothyroidism, hyperadrenocorticism), (5) not receiving or having received any medications known to affect lipid metabolism (eg, glucocorticosteroids) for at least 3 months before inclusion in the study, and (6) being fed diets not labeled as “low‐fat.”^1^

The healthy dogs belonged to students and staff of the College of Veterinary Medicine and Biomedical Sciences at Texas A&M University. Blood samples were collected from these dogs after food had been withheld for at least 12 hours. Blood samples were collected into glass tubes without additives, allowed to clot for 20 minutes, centrifuged, and serum aliquots were stored at −80°C until analysis. Owners were asked to complete a standardized questionnaire for each dog. Questions covered date of birth, sex and neuter status, body weight and BCS, current diet, current medications, and current and past health history of the dogs. Questionnaires from all dogs were reviewed to determine whether the dogs fit the inclusion criteria for the study.

### 2.2 Ethics approval

The study protocol was reviewed and approved by the Clinical Research Review Committee at Texas A&M University (TAMU‐CRRC# 2008‐37). The owners of each healthy dog enrolled in the study signed an informed owner consent form. For dogs with pancreatitis, only residual serum was used and therefore informed consent was not obtained.

### 2.3 Assays

Serum triglyceride (reference interval, 26‐108 mg/dL) and cholesterol (reference interval, 124‐335 mg/dL) concentrations were measured and serum biochemistry profiles were performed using analytically validated automated enzymatic assays (Roche/Hitachi MODULAR ANALYTICS D 2400 module, Roche Diagnostics, Indianapolis, Indiana). Serum Spec cPL concentration (reference interval, ≤200 μg/L) was measured using an analytically validated immunoassay described elsewhere.^17^ Serum total T4 concentration was measured using a solid‐phase chemiluminescent competitive assay (Immulite 2000 Canine Total T4, Siemens Healthcare Diagnostics, Deerfield, Illinois). Serum free T4 concentration was measured using a commercial equilibrium dialysis radioimmunoassay (Free T4 [by equilibrium dialysis], Antech Diagnostics, Irvine, California). Serum cTSH concentration was measured using a solid‐phase, 2‐site chemiluminescent immunometric assay (Immulite 2000 Canine TSH, Diagnostic Products Corporation, Los Angeles, California).

### 2.4 Lipoprotein profile analysis

Lipoprotein profiling was carried out using a bismuth sodium ethylenediaminetetraacetic acid (NaBiEDTA) density gradient ultracentrifugation method as previously described.^19^ This method has been validated in dogs^20^ and used to evaluate lipoprotein profiles in healthy dogs^21^ and in dogs with idiopathic hyperlipidemia^21^ and exocrine pancreatic insufficiency.^20^ This method also has been used to evaluate lipoprotein profiles in cats with hepatic lipidosis.^22^ The sodium salt of BiEDTA has been described as a novel solute forming a self‐generating density gradient during ultracentrifugation of serum samples for the separation of lipoproteins.^23^

The methodology used in our study previously was shown to identify 11 distinct lipoprotein fractions in dogs based solely on density characteristics.^20^, ^21^ Because the functional characteristics and composition of most lipoprotein density subfractions in dogs currently are unknown, all density subfractions can only be nominally assigned to traditional functional classes such as low‐density lipoproteins (LDL) or high‐density lipoproteins (HDL). Thus, density subfraction data are reported as previously described density ranges identified as R1 to R11 where: R1 (*d* < 1.017 g/mL), R2 (*d* = 1.019‐1.023 g/mL), R3 (*d* = 1.023‐1.029 g/mL), R4 (*d* = 1.029‐1.039 g/mL), R5 (*d* = 1.039‐1.050 g/mL), R6 (*d* = 1.050‐1.063 g/mL), R7 (*d* = 1.063‐1.091 g/mL), R8 (*d* = 1.091‐1.110 g/mL), R9 (*d* = 1.110‐1.133 g/mL), R10 (*d* = 1.133‐1.156 g/mL), and R11 (*d* = 1.156‐1.179 g/mL).^20^, ^21^ Based on a previously published classification, and based solely on their density characteristics, these fractions could be classified as: triglyceride‐rich lipoproteins (TRL; chylomicrons and very low‐density lipoproteins [VLDL]; *d* < 1.017 g/mL), LDL_1_ (*d* = 1.019‐1.023 g/mL), LDL_2_ (*d* = 1.023‐1.029 g/mL), LDL_3_ (*d* = 1.029‐1.039 g/mL), LDL_4_ (*d* = 1.039‐1.050 g/mL), LDL_5_ (*d* = 1.050‐1.063 g/mL), HDL_2b_ (*d* = 1.063‐1.091 g/mL), HDL_2a_ (*d* = 1.091‐1.110 g/mL), HDL_3a_ (*d* = 1.110‐1.133 g/mL), HDL_3b_ (*d* = 1.133‐1.156 g/mL), and HDL_3c_ (*d* = 1.156‐1.179 g/mL), respectively.^20^, ^21^ The above‐mentioned previously reported classification scheme for lipoprotein subfractions was used in our study.^20^, ^21^

### 2.5 Statistical analysis

Commercial statistical software packages were used for all statistical analyses (SPSS 16.0, SPSS Inc, Chicago, Illinois; Prism5, GraphPad, San Diego, California; R, http://www.r-project.org/). Data were analyzed for normal distribution using the Shapiro‐Wilk test. Summary statistics for continuous variables are reported as means (±SD) for parametric data and medians and ranges for nonparametric data. Categorical data are presented as counts (n) and percentages.

Normally distributed data were analyzed using *t* tests, whereas non‐normally distributed data were analyzed using Mann‐Whitney tests. Proportions were compared between groups using Fisher's exact tests with calculation of the odds ratio (OR) and the 95% confidence interval (95% CI) to test the possibility of an association between categorical variables. Sliced inverse regression or logistic regression analysis was used to test whether a relationship existed between group and lipoprotein profiles. Significance was set at *P* < .05 for all analyses.

## 3 RESULTS

### 3.1 Group 1 (dogs with pancreatitis)

Twenty‐eight dogs with a clinical diagnosis of pancreatitis initially were evaluated for inclusion in the study. Of these 28 dogs with pancreatitis, 11 had concurrent diseases or were being treated with drugs that might affect lipid metabolism and thus were excluded from the study. Specifically, 5 dogs had been diagnosed, treated or both for diabetes mellitus, 3 dogs had been diagnosed and treated for hypothyroidism, 1 dog had been diagnosed with hyperadrenocorticism and protein‐losing nephropathy, 1 dog had severe protein‐losing enteropathy, and 1 dog was being treated with prednisone. Therefore, Group 1 consisted of 17 dogs with a clinical diagnosis of pancreatitis that did not have any other recognized risk factors for hyperlipidemia. The characteristics of the dogs are shown in Table 1. These dogs belonged to 10 breeds and 2 dogs were mixed‐breed.

### 3.2 Group 2 (healthy dogs)

Fifty‐three healthy dogs were enrolled in Group 2 as controls. The characteristics of these dogs are shown in Table 1. Group 2 dogs belonged to 20 different breeds (34 dogs), and 19 dogs were mixed‐breed. Twenty‐six dogs were female (23 spayed) and 27 were male (23 castrated).

### 3.3 Serum triglyceride and cholesterol concentrations

Twelve of the 28 dogs (43%) with pancreatitis initially considered for enrollment in Group 1 had increased serum concentrations of triglycerides, cholesterol, or both. After excluding the 11 dogs with concurrent diseases that might affect lipid metabolism, 5 of 17 dogs (29%) in Group 1 had increased serum concentrations of triglycerides, cholesterol, or both. In contrast, only 5 of the 53 healthy dogs (9%) had increased serum concentrations of triglycerides, cholesterol, or both. This difference not significant (OR, 4; 95% CI, 0.9946‐16.09; *P* = .06).

**Figure 1:**
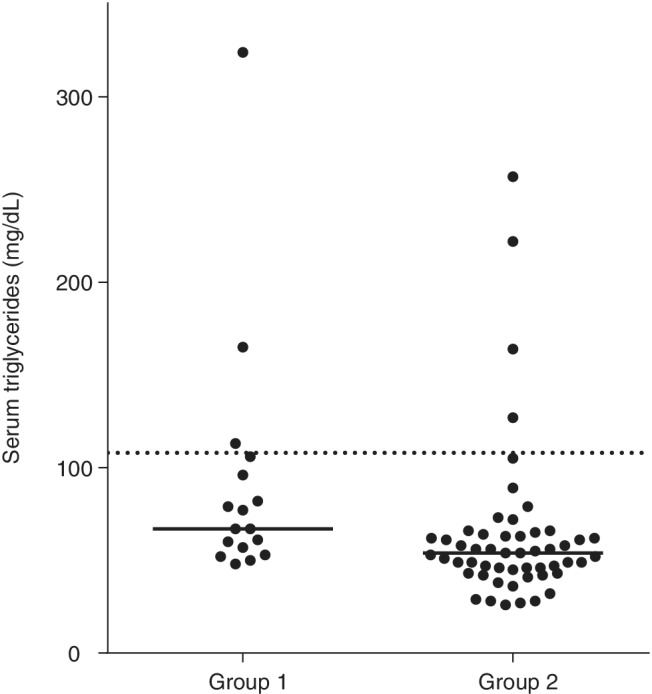
Serum triglyceride concentrations in 17 dogs with pancreatitis (Group 1) and 53 healthy control dogs (Group 2). Dogs in Group 1 had significantly higher (*P* = .002) serum triglyceride concentrations than dogs in Group 2. However, the majority of dogs in both groups had serum triglyceride concentrations within the reference interval. Hypertriglyceridemia, when present, was mild. The dashed line represents the upper limit of the reference interval (108 mg/dL) and the solid line represents the median

In Group 1, 3 of the 17 dogs (18%) had hypertriglyceridemia (Figure 1). Hypertriglyceridemia was mild (<350 mg/dL) in all 3 cases. Of the dogs in Group 2, 4 (7.5%) had hypertriglyceridemia, which was mild in all 4 cases (<300 mg/dL). No significant difference was found in the proportion of dogs that had hypertriglyceridemia between dogs with pancreatitis (18%) and healthy control dogs (7.5%; OR, 2.63; 95% CI, 0.52‐13.14; *P* = .35). However, a significant difference was found in serum triglyceride concentrations between Group 1 (median, 67 mg/dL; range, 48‐324 mg/dL) and Group 2 (median, 54 mg/dL; range, 26‐257 mg/dL; *P* = .003; Figure 1). However, as mentioned above, serum triglyceride concentrations were within the reference interval in the majority of dogs in both groups.

**Figure 2:**
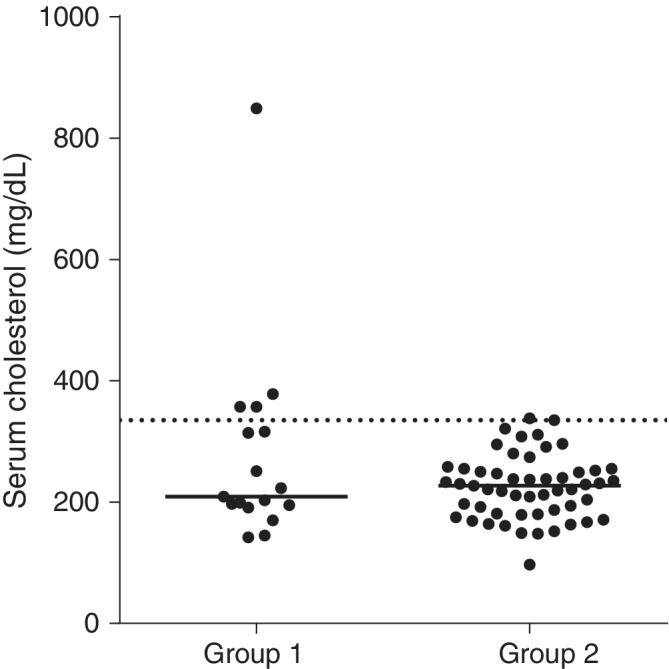
Serum cholesterol concentrations in 17 dogs with pancreatitis (Group 1) and 53 healthy control dogs (Group 2). There was no statistically significant difference in serum cholesterol concentration between the two groups (*P* = .56). The majority of dogs in both groups had serum cholesterol concentrations within the reference interval, whereas a small number of dogs had a mildly increased serum cholesterol concentration. The dashed line represents the upper limit of the reference interval (335 mg/dL) and the solid line represents the median

Of the 17 dogs in Group 1, 4 (24%) had hypercholesterolemia. Of the 53 control dogs in Group 2, only 1 had hypercholesterolemia. A significant difference in the proportion of dogs that had hypercholesterolemia was found between dogs with pancreatitis (Group 1) and healthy control dogs (OR, 16; 95% CI, 1.64‐155.5; *P* = .01). However, no significant difference in serum cholesterol concentrations was found between Group 1 (median, 209 mg/dL; range, 142‐849 mg/dL) and Group 2 (median, 227 mg/dL; range, 97‐338 mg/dL; *P* = .56; Figure 2).

### 3.4 Lipoprotein profile analysis

**Figure 3:**
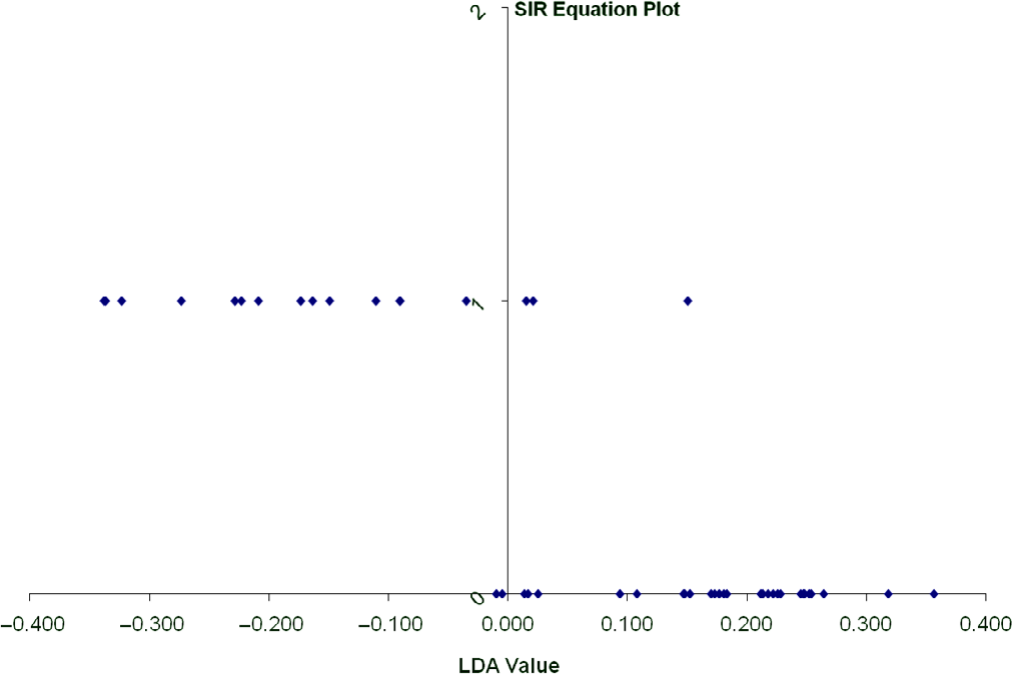
One dimensional sliced inverse regression plot showing classification of dogs into groups based on lipoprotein profile analysis. The vertical line represents the line that separates the two groups based on lipoprotein profile analysis. The linear discriminant analysis (LDA) value provides a ranking value for each dog. The dogs represented by the dots that are at the bottom of the graph are healthy (Group 2). The majority of dogs (except for 2 dogs) have a lipoprotein profile that plots to the right of the vertical line. The dogs represented by the dots at the top of the graph are dogs with pancreatitis (Group 1). The majority of these dogs (with the exception of 3) have a lipoprotein profile that is different from that of the healthy dogs. Sliced inverse regression analysis correctly classified approximately 90% of the dogs based on their lipoprotein profiles

**Figure 4:**
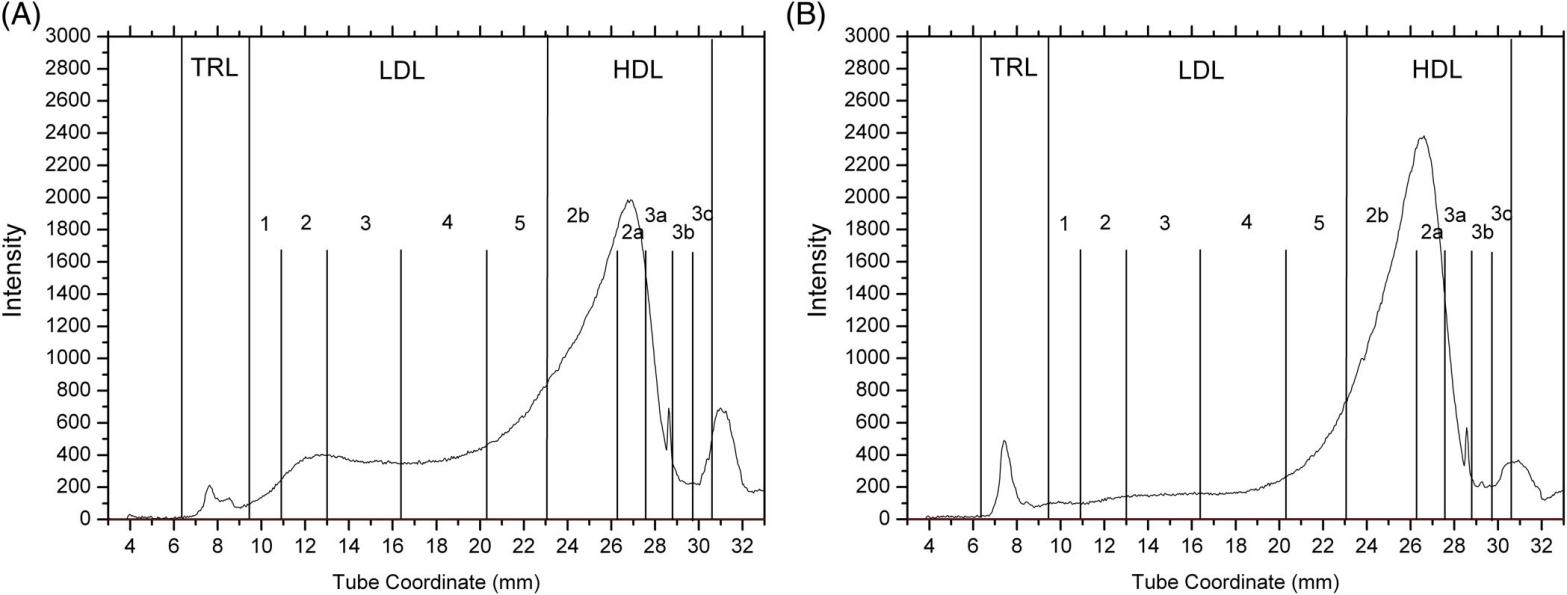
Lipoprotein density profiles from a representative dog with pancreatitis (A) and a representative healthy control dog (B). The dog in (A) shows profound increases in the nominal LDL fractions (mainly LDL_2_ through LDL_5_) compared to the healthy control dog shown in (B). Decreases in the TRL, HDL_2b_, and HDL_2a_ fractions might also be present in the dog in (A) compared to the dog in (B). Both dogs had serum triglyceride and cholesterol concentrations within their respective reference intervals. HDL, high‐density lipoproteins; LDL, low‐density lipoproteins; TRL, triglyceride‐rich lipoprotein

All 17 dogs of Group 1 were used for lipoprotein profile analysis. Age matched healthy control dogs from Group 2 (29 dogs) were selected for lipoprotein profile analysis. Sliced inverse regression analysis showed that lipoprotein profiles were distinctly different between Group 1 and Group 2 dogs (Eigenvalues, 0.6719; *R* ^2^ = 1.0; *P* = .001). Dogs could be classified as being healthy or having pancreatitis with 89% accuracy based on their lipoprotein profiles alone (Figure 3). The most important differences in the lipoprotein profiles between dogs with pancreatitis and healthy dogs involved increases in LDL_2_, LDL_3_, and LDL_4_, with less prominent decreases in TRL, HDL_2a_, and HDL_3c_ (Table 2). Figure 4A shows the lipoprotein density profile of a representative dog with pancreatitis. Figure 4B shows the lipoprotein density profile of a representative healthy dog. These results suggest that the main differences between these 2 representative lipoprotein profiles involved the LDL fractions, whereas the differences in TRL and HDL fractions were less prominent.

**Table 2:** Median integrated intensities of the regions of the ultracentrifugation tubes corresponding to 11 distinct density lipoprotein fractions based on density characteristics. Median integrated intensities in dogs with pancreatitis and healthy control dogs as well as the percent of total lipoproteins are displayed

Lipoprotein fraction	TRL	LDL1	LDL2	LDL3	LDL4	LDL5	HDL2b	HDL2a	HDL3a	HDL3b	HDL3c
Healthy	8096	4125	6867	13 836	23 190	45 529	142 200	53 279	12 554	7038	12 376
% of total lipoproteins	2.5	1.3	2.1	4.2	7	13.8	43.2	16.2	3.8	2.1	3.8
Pancreatitis	4931	3943	11 374	23 193	33 149	47 980	136 939	46 975	14 823	7383	8507
% of total lipoproteins	1.5	1.2	3.4	6.8	9.8	14.1	40.4	13.8	4.4	2.2	2.5
Direction of differences	↓	↓	↑	↑	↑	↑	↓	↓	↑	↑	↓

Abbreviations: HDL, high‐density lipoproteins; LDL, low‐density lipoproteins; TRL, triglyceride‐rich lipoproteins (includes chylomicrons and very‐low‐density lipoproteins).

## 4 DISCUSSION

The purpose of our study was to investigate serum lipid and lipoprotein abnormalities associated with naturally occurring pancreatitis in dogs. Mild increases in serum triglyceride or cholesterol concentrations or both were seen in a relatively small proportion of dogs with pancreatitis. The majority of dogs (>70%) with pancreatitis had serum triglyceride or cholesterol concentrations or both within the respective reference intervals. In contrast, important differences were identified in lipoprotein profiles between dogs with pancreatitis and healthy control dogs. Specifically, dogs with pancreatitis had higher LDL fractions (mainly LDL_2_, LDL_3_, and LDL_4_) and lower TRL and HDL fractions (mainly HDL_2a_ and HDL_3c_) than did healthy control dogs. These changes in lipoprotein profiles were evident in the majority of dogs with pancreatitis, even in cases in which serum triglyceride and cholesterol concentrations were within their respective reference intervals.

In the dogs investigated here, dogs with pancreatitis tended to more commonly have increased serum concentrations of triglycerides, cholesterol, or both (approximately 30%) than did healthy control dogs (approximately 10%), although this difference was not significant. Median serum triglyceride concentrations overall were higher in dogs with pancreatitis than in healthy dogs. However, this difference is clinically not important because the majority of dogs (>80%) had serum triglyceride concentrations within the reference interval and no significant difference was found in the proportion of dogs with hypertriglyceridemia between the 2 groups. In addition, hypertriglyceridemia was mild in all 3 dogs with pancreatitis that had hypertriglyceridemia. On the other hand, a higher proportion of dogs with pancreatitis had hypercholesterolemia (24%) compared to healthy dogs (1.9%). However, >75% of dogs with pancreatitis had normal serum cholesterol concentrations. Serum cholesterol concentrations were not significantly higher in dogs with pancreatitis, suggesting that hypercholesterolemia generally was mild in those cases, whereas the majority of dogs had normal serum cholesterol concentrations.

Our findings contradict a commonly held belief that pancreatitis commonly leads to increases in serum triglyceride or cholesterol concentrations or both in dogs. This belief is most likely based on clinical studies in dogs that show a relatively high prevalence of hyperlipidemia in dogs with pancreatitis.^11^, ^12^, ^13^ For example, in 1 study,^12^ 48 and 26% of dogs with pancreatitis were reported to have hypercholesterolemia and grossly lipemic serum, respectively. However, in those studies, dogs with secondary hyperlipidemia (eg, associated with diabetes mellitus or hypothyroidism) were not excluded from calculation of the prevalence of hyperlipidemia, and many dogs may have had hyperlipidemia as a result of diseases other than pancreatitis. This conclusion is supported by the findings of the present study. Before the exclusion of dogs that had secondary causes of hyperlipidemia (primarily diabetes mellitus and hypothyroidism), the overall prevalence of hyperlipidemia was 43%. However, when only dogs with pancreatitis and no other diseases were included, the prevalence of hyperlipidemia was much lower and it was mild in most cases.

Our study suggests that the majority of dogs (>70%) with pancreatitis have normal serum triglyceride and cholesterol concentrations, and only a small number (approximately 30% overall) have increases in serum triglyceride or cholesterol concentrations or both. Both hypertriglyceridemia and hypercholesterolemia were mild when present in dogs with pancreatitis (typically <200 and <400 mg/dL, respectively; Figures 1 and 2). Therefore, it is recommended that when moderate or severe increases in serum triglyceride or cholesterol concentrations or both are present in dogs with pancreatitis, other secondary causes of hyperlipidemia (eg, diabetes mellitus, hypothyroidism) or primary hyperlipidemia (eg, breed‐specific hyperlipidemias) be investigated as likely causes of hyperlipidemia.

Our findings are in agreement with those of studies of experimentally induced pancreatitis in dogs.^10^, ^15^, ^16^ Most of those studies have concluded that hypertriglyceridemia is not a consequence of experimentally induced pancreatitis in dogs.^10^, ^15^, ^16^ In 1 study, statistically significant increases in serum triglyceride concentrations were noted after induction of pancreatitis, but serum triglyceride concentrations remained within the reference interval throughout the study period.^16^ Similar findings were reported for hypercholesterolemia in those studies.^10^, ^15^, ^16^

Although major changes in serum triglyceride and cholesterol concentrations were not found in dogs with pancreatitis in our study, marked differences in lipoprotein profiles were found between dogs with pancreatitis and healthy controls. The dogs could be correctly classified in almost 90% of cases as having or not having pancreatitis based on their lipoprotein profile alone. The main changes in the lipoprotein profiles involved increases in the LDL fractions (mainly LDL_2_, LDL_3_, and LDL_4_). Decreases in HDL fractions such as HDL_2a_, HDL_3c_, and TRL also were present. Both LDL and HDL fractions contain mainly cholesterol.^1^ The fact that serum total cholesterol concentration was normal in the majority of dogs with pancreatitis might be explained by the fact that increases in LDL fractions were balanced by concurrent decreases in the HDL fractions. It is possible that during pancreatitis, lipoprotein metabolism is altered in a way that facilitates redistribution of lipid components (mainly cholesterol) between the LDL and HDL fractions. Further studies are needed to elucidate the mechanism of lipoprotein metabolism alterations in dogs with pancreatitis.

The changes in lipoprotein profiles in dogs with pancreatitis described here are in agreement with the findings of studies of experimentally induced pancreatitis in dogs.^10^, ^15^, ^16^ Those studies all have reported similar findings, including increases in LDL and decreases in HDL fractions. Based on these findings, serum alterations in the major lipoprotein fractions are similar between naturally occurring and experimentally induced pancreatitis.

Overall, TRL were decreased in dogs with pancreatitis in our study. To our knowledge, this finding has not been described previously in dogs with pancreatitis without concurrent diseases that affect lipoprotein metabolism. In healthy dogs, TRL is the main triglyceride‐containing lipoprotein fraction. Dogs have been reported not to have a cholesteryl ester transfer protein (CETP) that acts to selectively enrich LDL with cholesterol transferred from HDL,^24^ and so it is questionable that the particles in the nominal LDL density range are cholesteryl ester rich. These particles are likely to contain triglyceride and be closer in metabolic nature to TRL remnants. We did not determine the lipid composition of particles in the nominal LDL density range, but similarity to remnant lipoproteins would be compatible with the finding that serum triglyceride concentrations were significantly higher in dogs with pancreatitis. Further studies are needed to characterize the lipid content of the lipoprotein fractions of dogs with pancreatitis.

Overall, some of the changes observed in our study with regard to serum lipid concentrations and lipoprotein profiles are similar to the changes reported in humans or animal models with various types of inflammation or infection.^25^, ^26^, ^27^ Major changes take place in lipoprotein fractions and metabolism in response to inflammation, affecting the concentration, structure, and function of serum lipoproteins and mainly are mediated by hormones and cytokines such as tumor necrosis factor‐α (TNF‐α), interleukin‐1 (IL‐1), and IL‐6.^25^, ^26^, ^27^, ^28^, ^29^ Typical lipoprotein changes in response to acute or chronic inflammation in humans include decreases in HDL, whereas LDL fractions and triglycerides are more variable and may show increases, decreases, or remain unchanged.^25^, ^26^, ^27^, ^28^, ^29^ If the lipoproteins in the nominal LDL density range are indeed metabolically similar to TRL remnants, they could contribute to the inflammatory state.^30^ In most reports, serum triglyceride concentrations tend to increase, whereas serum LDL concentrations tend to decrease, with the exception of small LDL molecules.^25^, ^26^, ^27^, ^28^, ^29^ These changes in lipids and lipoproteins that occur during inflammation are part of the innate immune response and are believed to benefit the host through various mechanisms such as redistribution of nutrients to immune cells and binding of endotoxins and other biological substances.^29^, ^31^

Similar changes were observed in our study with respect to HDL and triglycerides. Inflammation in humans appears to induce a shift in LDL profiles leading to the appearance of small dense LDL molecules that correspond to fractions LDL_4_ and LDL_5_, a finding that was observed also in the dogs of Group 1 in our study.^25^, ^26^, ^27^, ^28^, ^29^ It seems likely that, as in humans, changes in serum lipid concentrations and lipoprotein profiles in dogs with pancreatitis might be the result of a general inflammatory response rather than a pancreatitis‐specific change.

Our study had some limitations. The major limitation is related to the small number of dogs with pancreatitis included in our study. This was partly a consequence of the strict inclusion criteria of our study that required exclusion of dogs that had evidence of concurrent diseases or conditions that can affect lipid metabolism. Previous studies have shown that pancreatitis and endocrine diseases that affect lipid metabolism commonly coexist.^11^, ^12^, ^13^, ^32^ In accordance with those studies, approximately 40% of the dogs initially enrolled in our study eventually were excluded because of the presence of concurrent disease that could have affected lipid metabolism. Another limitation was that the diagnosis of pancreatitis ideally should have been based on a combination of diagnostic tests in addition to clinical presentation and Spec cPL concentration. However, serum Spec cPL concentration has been shown to be very specific for pancreatitis.^33^, ^34^, ^35^ Although confirmation of the presence of pancreatitis usually requires histopathology, it is not commonly used in a clinical setting. Finally, the severity of pancreatitis in the dogs included in our study was not evaluated. It is likely that different degrees of severity corresponding to different degrees of inflammation might lead to differences in the degree of hyperlipidemia, although this has not been proven.

In conclusion, the majority of dogs with naturally occurring pancreatitis in our study (>70%) had serum triglyceride and cholesterol concentrations within their respective reference intervals. In the relatively small percentage of dogs that had increases in serum triglyceride or cholesterol concentrations or both, those increases generally were mild. Therefore, marked increases in serum triglyceride or cholesterol concentrations or both in dogs with pancreatitis might be unlikely to be the result of pancreatitis, and warrant further diagnostic investigation. In sharp contrast, important differences were identified in lipoprotein profiles between dogs with pancreatitis and healthy control dogs. Dogs with pancreatitis had higher nominal LDL fractions (mainly LDL_2_, LDL_3_, and LDL_4_) and lower TRL and HDL fractions (mainly HDL_2a_ and HDL_3c_) than did healthy control dogs. These changes in lipoprotein profiles were evident in the majority of dogs with pancreatitis, even in cases in which serum concentrations of triglyceride and cholesterol were normal. Further studies are needed to elucidate the mechanisms responsible for the changes in lipoprotein metabolism in dogs with pancreatitis. Also, additional research is required to determine whether these changes are specific for pancreatitis or a consequence of inflammation.

## CONFLICT OF INTEREST DECLARATION

Authors declare no conflict of interest.

## OFF‐LABEL ANTIMICROBIAL DECLARATION

Authors declare no off‐label use of antimicrobials.

## INSTITUTIONAL ANIMAL CARE AND USE COMMITTEE (IACUC) OR OTHER APPROVAL DECLARATION

Authors declare no IACUC or other approval was needed.

## HUMAN ETHICS APPROVAL DECLARATION

Authors declare human ethics approval was not needed for this study.
